# Granulomatous Disorder With Pulmonary and Renal Involvement: A Diagnostic and Therapeutic Dilemma

**DOI:** 10.7759/cureus.23149

**Published:** 2022-03-14

**Authors:** Merina Khan, Nida Saleem, Syed Nayer Mahmud, Muhammad Haneef, Hadia Bukhari

**Affiliations:** 1 Nephrology, Shifa International Hospital Islamabad, Islamabad, PAK

**Keywords:** anti-tuberculous therapy, anca vasculitis, mycophenolate mofetil, tuberculosis, granulomatosis with polyangiitis

## Abstract

Granulomatosis with polyangiitis (GPA) can present with a wide array of clinical signs and symptoms; therefore, it should be differentiated from other mimicking clinicopathological entities. We report a case of a 66-year-old gentleman who was found to have a mediastinal mass and histopathological examination showed chronic necrotizing granulomatous inflammation. The patient was managed on lines of pulmonary tuberculosis for 12 months and remained in remission for two years. Later, workup showed cytoplasmic antineutrophil cytoplasmic antibodies (c-ANCA)-associated granuloma with marked renal impairment, which responded to immunosuppression. From this, we suggest that in a patient with radiological evidence of mediastinal mass, the remote possibility of GPA must be kept in mind.

## Introduction

Granulomatosis with polyangiitis (GPA) is a small and medium-sized vessels vasculitis associated with positive antineutrophil cytoplasmic antibodies (ANCA), which causes necrotizing granulomatous inflammation mainly in the upper and lower respiratory tract in up to 85% of cases [1].

The association of pulmonary tuberculosis (TB) with GPA is uncommon. To definitely establish the etiology when these two differential diagnoses are considered is challenging, provided they share many similar clinical, radiological, and histopathological features. The cause of the diagnostic dilemma is that the presentation of GPA is heterogeneous in terms of severity, the number of organs involved, and the tempo of diseases. ANCA screening is considered highly specific for associated vasculitis, which includes GPA, eosinophilic granulomatosis with polyangiitis (EGPA), and microscopic polyangiitis [2]. Multiple other conditions besides ANCA-associated vasculitis (AAV) have the capability to induce ANCAs. These include infections such as TB [1], respiratory tract infections, endocarditis, malaria, and leprosy. Medications that can induce ANCAs include propylthiouracil, minocycline, isoniazid, and penicillamine [1].

TB and GPA display similar overlapping pulmonary and extrapulmonary features, including constitutional symptoms, arthralgias, cavitary lung lesion, malaise, and weight loss. Also, the pulmonary necrotizing granuloma in GPA can be difficult to distinguish radiologically from that of TB. Due to overlapping clinical features but drastically different management strategies required for TB and GPA, an extensive systematic and multidisciplinary radiological, histopathological, microbiological, and serological workup is required to ascertain the diagnosis and appropriate treatment [2]. There are limited data on the use of mycophenolate mofetil (MMF) for cytoplasmic ACNA (c-ANCA) vasculitis treatment [3,4]. Hence, we report a case of vasculitis diagnosed and treated as TB initially, which on subsequent relapse was worked up again, and was diagnosed as c-ANCA vasculitis, and was treated successfully with MMF.

## Case presentation

**Figure 1:**
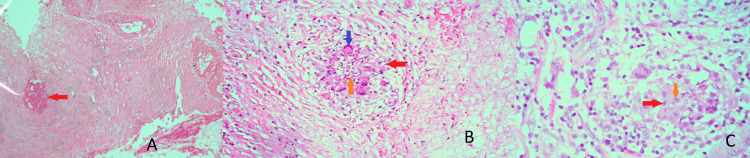
Hematoxylin and eosin-stained light microscopic images. A (x10); B and C (x40). Non-caseating granuloma (red arrow), necrosis (orange arrow), and epithelioid histiocytes (blue arrow).

A 66-year-old gentleman with no known comorbidities, who was an ex-smoker, presented to the pulmonology clinic with two months history of low-grade fever, anorexia, documented weight loss of 15 kg, night sweats, and malaise. After a thorough clinical history, high suspicion of either TB or malignancy was made. His workup started with relevant findings of raised erythrocyte sedimentation rate (ESR) of 102 mm/hour, and a CT scan of the chest showed a mediastinal mass encasing the arch vessels in the left superior mediastinum. Biopsy of the lesion showed chronic necrotizing granulomatous inflammation. He was then started on anti-tuberculous therapy (ATT). After which, he developed ATT-induced hepatitis; therefore, he was switched to modified ATT. Later, due to strong suspicion of malignancy, a left thoracotomy and redo biopsy were performed. Biopsy findings were consistent with chronic necrotizing granulomatous inflammation with no caseation, and malignant findings. Light microscopic images are shown in Figure 1.

**Figure 2:**
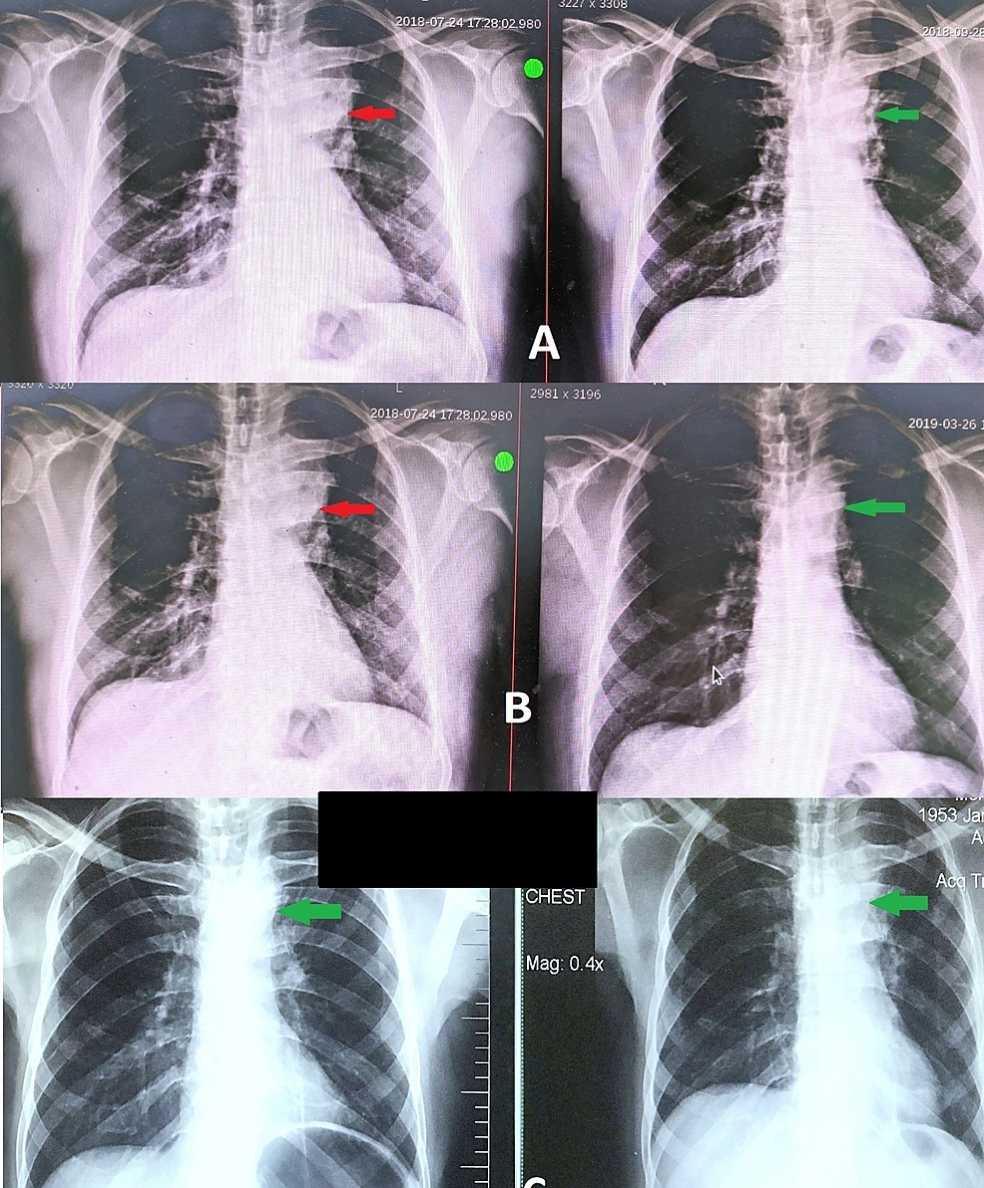
Chest X-ray images. A: Round ill-defined opacity in the mediastinum (red arrow) and reduced opacity size post-anti-tuberculous therapy initiation (green arrow). B: Recurrence of mediastinal opacity two years later (red arrow) and resolution of opacity post-mycophenolate mofetil (MMF) initiation (green arrow). C: Resolved opacity immediately post-MMF initiation (green arrow) and sustained resolution till now (green arrow).

Mycobacterium tuberculosis (MTB) DNA by polymerase chain reaction (PCR) was negative in the biopsy specimen. However, as there were foci of necrosis and moderate lymphocytic and neutrophilic infiltrates, therefore, ATT was continued for 12 months. Satisfactory response of clinical resolution of symptoms, weight gain of 29 kilograms, and resolution of mass lesion on imaging was achieved. Besides that, routine workup revealed 2+ blood in urinalysis, raised ESR, and elevated serum creatinine of 1.61 mg/dL. The patient was referred to the nephrology department, but he failed to comply with advice. Later on, after two years, he again presented with similar complaints in the pulmonology clinic, from where he was referred to a nephrologist before restarting ATT due to raised serum creatinine of 2.08 mg/dl, evidence of microscopic hematuria on urinalysis, and elevated serum uric acid of 10 mg/dl. Besides this, the patient also complained of arthralgias and bilateral pedal swelling for the last two weeks. There was a recurrence of mass in the left upper lung zone with mediastinal widening on repeat chest CT. After ruling out urological causes of hematuria, he was referred to a rheumatologist with a high index of suspicion of vasculitis. Autoimmune workup showed positive c-ANCA and negative rheumatoid factor, anti-cyclic citrullinated peptide (CCP) antibodies, and serum angiotensin-converting enzyme (ACE) levels. To treat c-ANCA-associated vasculitis, he was counseled regarding the initiation of intravenous cyclophosphamide, but the patient refused due to possible side effects. Similarly, azathioprine was not started as the patient was already on allopurinol for his raised serum uric acid levels. Hence, pulse steroids and MMF were started. The patient had close follow-ups with a rheumatologist and nephrologist. He dramatically improved clinically. Moreover, there was a complete radiological resolution of the mass lesion. His serum creatinine got stabilized at 1.4 mg/dL and his ESR dropped down from 102 mm/hour to 12 mm/hour. Figure 2 summarizes chest X-ray images during the entire course of illness and response to therapy as shown below.

## Discussion

The above-discussed case exemplifies the difficulty in distinguishing between TB and GPA, given their similar clinical, radiological, and histopathological features, with added complexity in this instance with initially good response to ATT and resolution of the mass lesion.

Similarities between the two conditions highlighted by this case include the presence of a cavitary lesion, necrotizing granulomatous inflammation on biopsy, and a positive proteinase-3 (PR3) ANCA. To differentiate the two conditions, other diagnostic modalities are needed to be considered, including lung biopsy, sputum analysis, and thorough clinical history [5].

The Wegener’s triad of necrotizing glomerulonephritis, necrotizing granulomatous inflammation of the respiratory tract, and necrotizing systemic angiitis give the clue about vasculitis [1,2]. This disease has a peak incidence in the fourth and fifth decades. Misdiagnosis and subsequently inappropriate treatment will result in worsening of underlying primary pathology. Renal disease is estimated to occur in 11-18% of cases at presentation and 77-85% of cases during the disease course, with extra-renal manifestation often preceding the renal disease. Initial and recurrent renal damage may lead to chronic renal insufficiency in up to 42% of patients. There are some non-specific laboratory abnormalities like leukocytosis, thrombocytosis, elevated ESR, and anemia, which are typical features of all inflammatory conditions. Organ-specific laboratory evaluations include urinalysis, urinary sediment, and serial creatinine. The sensitivity of PR3 ANCA is about 90% in active GPA and 40% when the disease is in remission. The specificity of PR3 ANCA in the diagnosis of GPA exceeds 95% [6].

As in our case, there were overlapping findings and clinical presentation of both TB and vasculitis, but TB being more prevalent in our region and a good response to ATT, both were favoring the diagnosis of TB. However, the second attack of vasculitis with a similar presentation after two years created the surge for re-evaluation and subsequent testing for GPA. Therefore, it is very important to always consider diseases, presenting with similar features, as differentials. Delayed diagnosis of GPA has significant long-term risks and may have unfavorable outcomes. Also, inappropriately starting immunosuppression in such a patient without ruling out other possible differentials of similar manifestations might even result in worsening of underlying TB and even precipitate into miliary TB [1,2]. Table 1 summarizes the commonalities and differentiating features of TB and GPA.

**Table 1:** Comparison between tuberculosis and granulomatosis with polyangiitis. ANCA: antineutrophil cytoplasmic antibodies; ESR: erythrocyte sedimentation rate; ZN: Ziehl-Neelsen; MTB PCR: Mycobacterium tuberculosis polymerase chain reaction; p-ANCA: perinuclear ANCA; c-ANCA: cytoplasmic ANCA.

Similarities between tuberculosis and granulomatosis with polyangiitis		
	Tuberculosis	Granulomatosis with polyangiitis
Constitutional symptoms (fever, malaise, weight loss, night sweats, arthralgias)	Very common	Very common
Hemoptysis	Common in tracheobronchial tuberculosis	Very common
Ocular manifestations (uveitis, keratitis)	Common	Very common
ANCA positivity	Present in some cases	Very common
Laboratory findings (anemia, leukocytosis, raised ESR)	Common	Common
Pulmonary cavitary lesion on CT scan	Very common	Very common
Necrotizing granulomatous inflammation on histopathology	Very common	Very common
Differences between tuberculosis and granulomatosis with polyangiitis		
	Tuberculosis	Granulomatosis with polyangiitis
Exposure history	Significant	Insignificant
Nasal involvement (epistaxis, crusting, nasal deformity)	Uncommon	Very common
Oral manifestations (gingivitis, mucosal ulceration, cobblestoning of buccal mucosa)	Very rare	Common
Renal manifestations ( hematuria, proteinuria, renal impairment)	Uncommon	Very common
Necrotizing glomerulonephritis	Very rare	Very common
Necrotizing leukocytoclastic vasculitis	Extremely rare	Common
Granuloma	Caseating mostly	Non-caseating
p-ANCA/myeloperoxidase ANCA positivity	In 75% of ANCA-positive cases [7]	Rare
c-ANCA/proteinase-3 ANCA positivity	In 12.5% of ANCA-positive cases [7]	80-90% positivity
Sputum analysis (ZN staining, culture, MTB PCR)	Positive	Negative
Interferon gamma release assay, skin prick test	Positive	Negative

From this case, we can suggest that patient might have a pre-existing misdiagnosed GPA due to histopathological evidence of non-caseating granuloma, and negative MTB PCR on the mass biopsy specimens. Moreover, evidence of renal impairment and hematuria are suggestive of GPA. However, spontaneous remission of vasculitis and ATT responsive coexistent TB with vasculitis are the additional possibilities in this case.

There are emerging data regarding the role of MMF in the acute management of c-ANCA-positive vasculitis [3]. One recent trial has reported a non-inferior role of MMF in comparison to cyclophosphamide in achieving disease remission, but with more frequent relapses [8]. Currently, MMF is recommended as a second-line maintenance immunosuppressive therapy [4] in patients with c-ANCA vasculitis, when azathioprine is contraindicated, as in our case, the patient had hyperuricemia. From the excellent response of our patient to MMF induction therapy, it can be suggested that in a patient who is not willing for intravenous cyclophosphamide and rituximab, MMF can be tried as an acute management strategy, even in c-ANCA-positive vasculitis.

## Conclusions

It is of utmost importance to have a high index of suspicion to ensure that the exact diagnosis is made, and TB must be considered a possible differential or coexistent pathology in granulomatous disorders. As there are no reliable pathognomonic imaging features that clinch the diagnosis, therefore, it is imperative to identify certain patterns of disease that are suggestive of GPA. The constellation of relevant clinical history with supportive laboratory and radiological findings is necessary for the establishment of accurate diagnosis and specific treatment. In a patient with clinical suspicion of coexistent TB and GPA, ATT can be started, as it can probably contribute to the resolution of pulmonary granuloma. A successful treatment response with MMF exemplifies that it is mandatory to keep our vision and options tailored to specific patient needs and symptomatology to provide more effective treatment options.

## References

[1] Mahmood FS, Schwatz E, Kurrup S, Sharp C, Hands G, Moody A (2013). A diagnostic dilemma: differentiating between granulomatosis with polyangiitis and tuberculosis. Clin Med (Lond).

[2] Pandhi N, Kajal NC, Nagaraja CL, Swathi K, Prabhudesai R (2015). Wegener's granulomatosis disease mimicking pulmonary tuberculosis. J Assoc Chest Physicians.

[3] Iatrou C, Zerbala S, Revela I, Spanou E, Marinaki S, Nakopoulou L, Boletis J (2009). Mycophenolate mofetil as maintenance therapy in patients with vasculitis and renal involvement. Clin Nephrol.

[4] Langford CA, Talar-Williams C, Sneller MC (2004). Mycophenolate mofetil for remission maintenance in the treatment of Wegener's granulomatosis. Arthritis Rheum.

[5] Molinari L, Melamud JI, Ferrari L, Landi P, Semeniuk G, Quadrelli SA (2009). Wegener's granulomatosis and tuberculosis. A bad combination. (Article in Spanish). Medicina (B Aires).

[6] Haridas V, Haridas K (2017). Granulomatosis with polyangiitis (GPA) mimicking tuberculosis. J Assoc Physicians India.

[7] Sherkat R, Mostafavizadeh K, Zeydabadi L, Shoaei P, Rostami S (2011). Antineutrophil cytoplasmic antibodies in patients with pulmonary tuberculosis. Iran J Immunol.

[8] Jones RB, Hiemstra TF, Ballarin J (2019). Mycophenolate mofetil versus cyclophosphamide for remission induction in ANCA-associated vasculitis: a randomised, non-inferiority trial. Ann Rheum Dis.

